# Discordance Between 16S rRNA Similarity and Genome‐Based Species Boundaries in *Gluconobacter*

**DOI:** 10.1002/mbo3.70335

**Published:** 2026-06-18

**Authors:** Shintaro Maeno, Naoya Kataoka, Minenosuke Matsutani, Toshiharu Yakushi

**Affiliations:** ^1^ Research Center for Thermotolerant Microbial Resources Yamaguchi University Yamaguchi Japan; ^2^ Organization for Research Initiatives Yamaguchi University Yamaguchi Japan; ^3^ Department of Food, Aroma and Cosmetic Chemistry, Faculty of Bioindustry Tokyo University of Agriculture Hokkaido Japan

**Keywords:** 16S rRNA sequence, *Gluconobacter*, microbiome, misclassification, taxonomy

## Abstract

The 16S rRNA gene is widely used for bacterial identification, but its resolving power is limited in several taxa. In the genus *Gluconobacter*, species boundaries remain unclear despite reliance on 16S‐based classification. To examine these limitations, we analyzed all publicly available complete genomes (*n* = 12) together with curated 16S rRNA sequences and compared 16S rRNA sequences similarity with genome‐based relatedness. Within the sampled complete genomes, 16S rRNA sequences were highly conserved and showed substantial overlap between intra‐ and interspecific similarities. In contrast, average nucleotide identity (ANI)‐based divergence was pronounced: ANI values ranged from 79.7% to 100%, and *G. oxydans* included more than one genomic lineage. Notably, two strains (H24 and 1.637) showed higher ANI values (97.2%–97.4%) to *G. thailandicus* than to other *G. oxydans* strains. Even the hypervariable V4, V6, and V8 regions lacked clade‐specific signatures, indicating limited species‐level resolution within this group. Furthermore, type‐strain 16S rRNA sequences of *G. oxydans*, *G. frateurii*, and *G. japonicus* show ≥ 99% similarity and are grouped within the same non‐redundant representative in the SILVA NR99 database, indicating limited species‐level resolution. These results demonstrate a clear mismatch between 16S rRNA sequences similarity and genomic structure in *Gluconobacter*. Genome‐based criteria, supported by refined reference databases and validated gene markers, are essential for accurate taxonomy and microbiome‐based analyses.

## Graphical abstract

16S rRNA sequences in *Gluconobacter* show high conservation with overlapping intra‐ and interspecific similarities, limiting species‐level resolution. In contrast, genome‐based ANI reveals clear divergence and distinct lineages, highlighting a fundamental discordance between 16S rRNA sequence‐based classification and genome‐level relationships.

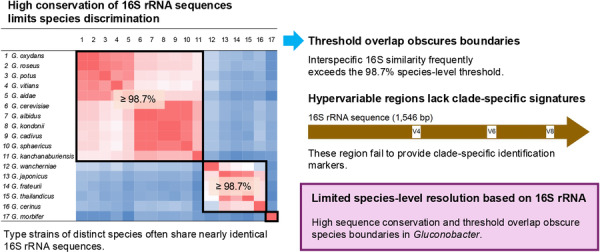

## 1 Introduction

The 16S rRNA gene is the most widely used molecular marker in bacterial taxonomy and microbiome research because of its universal conservation and mixture of variable and conserved regions (Clarridge 2004; Johnson et al. 2019). While powerful for genus‐level identification, its resolving power varies substantially among taxa, and closely related species often share nearly identical sequences that hinder species‐level discrimination (Chun et al. 2018; Church et al. 2020). These limitations are well‐documented in both clinical and environmental contexts, where reliance on 16S alone can lead to over‐classification or misidentification (Church et al. 2020; Tanno et al. 2022). Further complicating interpretation, many bacterial genomes carry multiple 16S rRNA operons, and intragenomic variation—even when subtle—can cause ASV collapse or inflate perceived diversity (Espejo and Plaza 2018; Tanno et al. 2022).

The genus *Gluconobacter* (*Acetobacteraceae*) comprises obligately aerobic acetic acid bacteria widely used in food fermentations and oxidative bioprocesses (Lynch et al. 2019). Despite their industrial importance, taxonomy within this genus has remained problematic for decades owing to the extreme conservation of 16S rRNA sequences among species (Schmitz et al. 2021; Sievers et al. 1995). In microbiome datasets, reads are frequently assigned as *G. oxydans* or *Gluconobacter* sp., even though recognized species such as *G. frateurii, G. cerinus*, and *G. japonicus* cannot be reliably separated by 16S alone. This limitation is also reflected in widely used microbiome analysis workflows, as represented by reference databases such as SILVA and RDP (Cole et al. 2014; Quast et al. 2013), where multiple type‐strain sequences share > 99% identity and are often represented within the same non‐redundant reference cluster, thereby obscuring species‐level boundaries (Schmitz et al. 2021). Studies using alternative markers—*recA*, *rpoB*, *adhA*, or multilocus sequence analysis—have demonstrated improved resolution (Lynch et al. 2019). Notably, early analyses of 16S–23S ITS regions had already suggested that several *Gluconobacter* species comprise multiple lineages requiring taxonomic reconsideration (Takahashi et al. 2006; Yamada and Yukphan 2008). In particular, systematic analyses linking intragenomic heterogeneity and genome‐level divergence remain lacking. Consequently, fundamental questions—such as how accurately 16S reflects species boundaries and how dependable current reference databases truly are—remain unanswered.

In this study, we quantitatively evaluated 16S rRNA gene conservation and diversity within currently available complete genomes of *Gluconobacter*. By integrating 16S rRNA‐based phylogenetic analysis with genome‐wide average nucleotide identity (ANI), we evaluated the limitations of 16S‐based classification and their implications for taxonomy and microbiome research.

## 2 Materials and Methods

### 2.1 16S rRNA Sequence Dataset

This study analyzed all complete genome sequences (*n* = 12) of the genus *Gluconobacter* available in the NCBI database as of October 28, 2025 (Supplementary Table S1). Complete genomes were intentionally selected because *Gluconobacter* species typically harbor multiple copies of the 16S rRNA gene, and draft assemblies frequently collapse or fragment ribosomal operons due to their repetitive structure. Accurate assessment of copy number and intragenomic heterogeneity, therefore, requires fully resolved genome assemblies. When multiple assemblies were available for the same strain, the most recently released RefSeq assembly was used. All genomes were re‐annotated using DFAST v1.6.0 (Tanizawa et al. 2018), and ribosomal RNA features were extracted from the resulting annotation files. The fix‐origin option was applied to standardize the sequence orientation from the *dnaA* gene. For genomes containing multiple 16S rRNA gene copies, loci were numbered sequentially from the upstream end.

To expand the comparative dataset beyond the genomes analyzed, 16S rRNA gene sequences of the type strains of 18 currently validated *Gluconobacter* species were retrieved from NCBI according to the List of Prokaryotic names with Standing in Nomenclature (LPSN) (Supplementary Table S2). In addition, 16S rRNA sequences annotated as *Gluconobacter* were extracted from the SILVA SSU Ref NR99 release 138.2 database (Glöckner et al. 2017), which is widely used for microbial community analyses. Sequences shorter than 1350 bp were removed using seqkit v2.5.1 (Shen et al. 2016). One entry, “Uncultured *Gluconobacter* sp.” (accession JQ061268), showed > 99% identity to *Leifsonia shinshuensis* by BLASTn and was therefore excluded as a misannotated sequence.

### 2.2 Genome‐Wide ANI Calculation

Pairwise average nucleotide identity (ANI) values among the 12 complete genomes were calculated using FastANI v1.33 (Jain et al. 2018) with default settings. ANI results were summarized as a pairwise similarity matrix (Table 1) and compared with 16S rRNA gene sequence identities to evaluate the concordance between genome‐scale relatedness and 16S rRNA‐based similarity.

**Table 1:** Average nucleotide identity (ANI) among 12 complete *Gluconobacter* genomes.

	Accession	Copy number of 16S rRNA genes	*G. sphaericus* SJF2‐1	*G. oxydans* DSM 3504	*G. oxydans* ATCC 9937	*G. oxydans* 621H	*G. roseus* B4931_cRAD	*G. albidus* TMW2.1191	*G. thailandicus* HD924	*G. oxydans* a H24	*G. oxydans* a 1.637	*G. japonicus* T12B	*G. cerinus* MSC4Y	*G. frateurii* ML. ISBL3
*G. sphaericus* SJF2‐1	GCF_016757795.1	4	100	90.8	90.6	90.6	87.2	87.0	80.7	80.6	80.0	80.1	80.1	79.9
*G. oxydans* DSM 3504	GCF_000583855.1	4	90.8	100	96.3	96.0	90.4	86.6	80.3	80.2	80.1	79.8	79.8	79.9
*G. oxydans* ATCC 9937	GCF_030450005.1	4	90.6	96.3	100	97.5	90.6	86.6	80.9	80.5	80.1	80.0	79.9	80.0
*G. oxydans* 621H	GCF_900175995.1	4	90.6	96.0	97.5	100	90.6	86.5	81.0	80.2	80.0	79.8	79.7	80.0
*G. roseus* B4931_cRAD	GCF_046058915.1	4	87.2	90.4	90.6	90.6	100	86.2	79.9	80.1	80.0	80.0	79.7	79.8
*G. albidus* TMW2.1191	GCF_002005485.1	5	87.0	86.6	86.6	86.5	86.2	100	80.5	80.6	79.8	80.3	80.4	79.9
*G. thailandicus* HD924	GCF_008118345.1	5	80.7	80.3	80.9	81.0	79.9	80.5	100	97.2	97.4	86.9	84.0	90.0
*G. oxydans* H24	GCF_000311765.1	5	80.6	80.2	80.5	80.2	80.1	80.6	97.2	100	100.0	87.0	84.2	89.9
*G. oxydans* 1.637	GCF_001680825.1	5	80.0	80.1	80.1	80.0	80.0	79.8	97.4	100.0	100	87.0	83.9	90.0
*G. japonicus* T12B	GCF_050500265.1	5	80.1	79.8	80.0	79.8	80.0	80.3	86.9	87.0	87.0	100	83.3	87.9
*G. cerinus* MSC4Y	GCF_048388395.1	5	80.1	79.8	79.9	79.7	79.7	80.4	84.0	84.2	83.9	83.3	100	83.8
*G. frateurii* ML.ISBL3	GCF_022453865.1	5	79.9	79.9	80.0	80.0	79.8	79.9	90.0	89.9	90.0	87.9	83.8	100

^a^ *G. oxydans* H24 and 1.637 showed 97.2%–97.4% ANI to *G. thailandicus* HD924 and are considered *G. thailandicus*‐related strains in this study.

### 2.3 Extraction, Alignment, and Identity Analysis of 16S rRNA Genes

All 16S rRNA gene sequences obtained from complete genomes were aligned using MAFFT v7.526 with the ‐‐auto option (Katoh and Standley 2013). Pairwise single‐nucleotide polymorphisms (SNPs) were calculated using snp‐dists v0.8.2 (https://github.com/tseemann/snp-dists). For pairwise identity calculations, global sequence identities were derived from the multiple sequence alignment as the proportion of identical nucleotides across aligned positions, excluding gap positions. This alignment‐based approach was used in place of BLAST‐based local identity estimates to provide a more accurate assessment of full‐length sequence similarity. To ensure taxonomically consistent comparisons, a separate dataset consisting of full‐length (or near full‐length) 16S rRNA gene sequences from type strains was constructed. These sequences were aligned using the same MAFFT settings, and pairwise global identities were calculated as described above. Because many sequences showed 100% identity, redundant sequences were removed using seqkit v2.5.1 (Shen et al. 2016). To evaluate sequence variability among the nine canonical variable regions (V1–V9), the *Escherichia coli* K‐12 MG1655 16S rRNA gene (GenBank accession U00096.3) was used as a positional reference (Chakravorty et al. 2007; McLaughlin et al. 1981).

### 2.4 Phylogenetic Analysis of 16S rRNA Sequences

A total of 75 unique 16S rRNA sequences, including *Acetobacter aceti* NCIB 8621 ^T^ (accession no. X74066) as an outgroup, were used for phylogenetic reconstruction. Maximum‐likelihood phylogenetic trees were constructed using IQ‐TREE v2.4.0 with the GTR + G substitution model (Nguyen et al. 2015). Branch supports were assessed using 100 bootstrap replicates. The best‐fit substitution model was selected using ModelFinder implemented in IQ‐TREE based on the Bayesian Information Criterion (BIC) (Kalyaanamoorthy et al. 2017). The resulting trees were visualized with iTOL v7 (https://itol.embl.de/).

## 3 Results

### 3.1 Genomic Overview and 16S rRNA Dataset Characteristics

The dataset comprised all publicly available complete genomes (*n* = 12) representing nine species of *Gluconobacter*, 16S rRNA gene sequences from 18 type strains, and an additional 111 sequences retrieved from the SILVA database. The sequences ranged from 1,350 to 1,493 bp in length (mean = 1,440.2 bp). After alignment with MAFFT, the total alignment length was 1,546 bp, covering the full‐length 16S rRNA gene region.

The 16S rRNA genes derived from complete genomes comprised four to five copies per genome (a total of 55 copies; Table 1). “16S ribosomal RNA” features annotated by DFAST were extracted. Across all strains, up to 33 SNPs were observed among 16S rRNA gene copies, whereas sequence variation among 16S rRNA gene copies within individual genomes was limited to 0–2 SNPs, indicating very low within‐genome heterogeneity (Supplementary Table S3). Notably, *G. oxydans* exhibited the highest level of intra‐species divergence within the genus, with up to 27–29 SNPs among its 16S rRNA sequences.

To further evaluate the genomic relatedness among *G. oxydans* strains, pairwise ANI values were calculated using FastANI. FastANI analysis revealed ANI values ranging from 79.7% to 100% among the 12 strains of *Gluconobacter* (Table 1). Based on the species‐level cutoff of 95% ANI (Chun et al. 2018; Riesco and Trujillo 2024), *G. oxydans* was subdivided into at least two distinct genomic clusters. Alignment fractions ranged from 31.9% to 85.1%, indicating that ANI values were calculated over substantial portions of the genome. Specifically, DSM 3504, ATCC 9937, and 621H shared 96.0%–100% ANI and formed a single cluster. In contrast, H24 and 1.637 showed 99.9% mutual identity but only ~80% ANI relative to this group. These two strains also exhibited higher ANI values (97.2%–97.4%) to *G. thailandicus* HD924 than to other *G. oxydans* strains. These values exceed commonly used species‐level thresholds, suggesting that H24 and 1.637 may be more closely related to *G. thailandicus* than to *G. oxydans* sensu stricto. Two major genomic groups were identified among the analyzed complete genomes. The first group, consisting of *G. sphaericus*, *G. oxydans*, *G. roseus*, and *G. albidus*, showed 86.2%–100% ANI, whereas the second group—comprising *G. thailandicus*, *G. japonicus*, *G. cerinus*, and *G. frateurii*—showed 83.3%–100% ANI. The latter group corresponded to “*Gluconobacter* clade I” previously reported (Matsutani et al. 2014). Within the genus, *Gluconobacter* clade I and *G. albidus* possessed five copies of the 16S rRNA gene per genome, whereas all other species carried four copies.

### 3.2 Sequence Identity Analysis of the 16S rRNA Gene

To provide a taxonomically consistent benchmark, we further examined pairwise 16S rRNA gene sequence identity among type strains representing validly named species (Table 2). These results provide a taxonomically controlled benchmark, which was further compared with the expanded dataset including additional reference sequences (Table 3). In this dataset, interspecific identity values ranged from 97.36% to 100%, with a substantial proportion of comparisons exceeding the commonly accepted 98.7% species‐level threshold. Notably, several species pairs, including *G. oxydans* and *G. roseus*, exhibited identical 16S rRNA gene sequences (100%), while many other interspecific comparisons remained above 99%. Even among more distantly related species, identity values frequently remained above 97%, indicating a narrow dynamic range of sequence divergence across the genus. These results indicate that even when analysis is restricted to type strains, representing taxonomically validated reference points, the 16S rRNA gene provides limited resolution for distinguishing species within the genus.

**Table 2:** Pairwise 16S rRNA gene sequence identity among species of the genus *Gluconobacter* type strains.

	*G. oxydans*	*G. roseus*	*G. potus*	*G. vitians*	*G. aidae*	*G. cerevisiae*	*G. albidus*	*G. kondonii*	*G. cadivus*	*G. sphaericus*	*G. kanchanaburiensis*	*G. wancherniae*	*G. japonicus*	*G. frateurii*	*G. thailandicus*	*G. cerinus*	*G. morbifer*
*G. oxydans*	**100**	**100**	**99.80**	**99.73**	**99.72**	**99.30**	**99.29**	**99.22**	**99.18**	**99.22**	**99.08**	98.37	98.08	98.04	97.91	98.07	97.51
*G. roseus*	**100**	**100**	**99.86**	**99.79**	**99.72**	**99.29**	**99.29**	**99.22**	**99.22**	**99.22**	**99.08**	98.37	98.08	97.94	98.01	98.01	97.51
*G. potus*	**99.80**	**99.86**	**100**	**99.66**	**99.72**	**99.16**	**99.15**	**99.08**	**99.12**	**99.08**	**99.08**	98.23	97.94	97.82	97.78	97.93	97.51
*G. vitians*	**99.73**	**99.79**	**99.66**	**100**	**99.50**	**99.09**	**99.08**	**99.01**	**99.05**	**99.01**	**99.15**	98.44	98.01	97.89	97.85	97.86	97.73
*G. aidae*	**99.72**	**99.72**	**99.72**	**99.50**	**100**	**99.29**	**99.29**	**99.22**	**99.22**	**99.22**	**99.22**	98.08	97.80	97.66	97.73	97.73	97.36
*G. cerevisiae*	**99.30**	**99.29**	**99.16**	**99.09**	**99.29**	**100**	**100**	**99.93**	**99.93**	**99.93**	**99.50**	98.23	97.94	97.84	97.82	98.12	97.87
*G. albidus*	**99.29**	**99.29**	**99.15**	**99.08**	**99.29**	**100**	**100**	**99.93**	**99.93**	**99.93**	**99.50**	98.23	97.94	97.80	97.87	98.08	97.87
*G. kondonii*	**99.22**	**99.22**	**99.08**	**99.01**	**99.22**	**99.93**	**99.93**	**100**	**100**	**99.86**	**99.43**	98.15	97.87	97.73	97.80	98.15	97.80
*G. cadivus*	**99.18**	**99.22**	**99.12**	**99.05**	**99.22**	**99.93**	**99.93**	**100**	**100**	**99.86**	**99.43**	98.15	97.87	97.76	97.71	98.20	97.81
*G. sphaericus*	**99.22**	**99.22**	**99.08**	**99.01**	**99.22**	**99.93**	**99.93**	**99.86**	**99.86**	**100**	**99.43**	98.15	97.94	97.80	97.87	98.08	97.87
*G. kanchanaburiensis*	**99.08**	**99.08**	**99.08**	**99.15**	**99.22**	**99.50**	**99.50**	**99.43**	**99.43**	**99.43**	**100**	97.87	97.58	97.44	97.52	97.59	97.65
*G. wancherniae*	98.37	98.37	98.23	98.44	98.08	98.23	98.23	98.15	98.15	98.15	97.87	**100**	**99.15**	**99.01**	**98.94**	98.44	97.51
*G. japonicus*	98.08	98.08	97.94	98.01	97.80	97.94	97.94	97.87	97.87	97.94	97.58	**99.15**	**100**	**99.86**	**99.79**	**99.29**	97.58
*G. frateurii*	98.04	97.94	97.82	97.89	97.66	97.84	97.80	97.73	97.76	97.80	97.44	**99.01**	**99.86**	**100**	**99.51**	**99.17**	97.44
*G. thailandicus*	97.91	98.01	97.78	97.85	97.73	97.82	97.87	97.80	97.71	97.87	97.52	**98.94**	**99.79**	**99.51**	**100**	**99.16**	97.37
*G. cerinus*	98.07	98.01	97.93	97.86	97.73	98.12	98.08	98.15	98.20	98.08	97.59	98.44	**99.29**	**99.17**	**99.16**	**100**	97.59
*G. morbifer*	97.51	97.51	97.51	97.73	97.36	97.87	97.87	97.80	97.81	97.87	97.65	97.51	97.58	97.44	97.37	97.59	**100**

*Values ≥ 98.7% (in bold) exceed the generally accepted species‐level threshold for 16S rRNA gene sequence identity.

**Table 3:** Pairwise 16S rRNA gene sequence identity among representative and reference sequences of *Gluconobacter*.

	No. ofsequences	*G. aidae*			*G. albidus*			*G. cadivus*			*G. cerevisiae*			*G. cerinus*			*G. frateurii*			*G. japonicus*			*G. Kanchan aburiensis*			*G. kondonii*		
*G. aidae*	1	100*																										
*G. albidus*	16	98.1	―	**99.3**	**98.8**	―	**100**																					
*G. cadivus*	1	**99.2**			**98.8**	―	**100**	**100**																				
*G. cerevisiae*	1	**99.2**			**98.8**	―	**100**	**99.9**			**100**																	
*G. cerinus*	18	96.7	―	97.7	95.7	―	98.2	96.8	―	98.2	96.8	―	98.1	97.3	―	**100**												
*G. frateurii*	20	96.5	―	97.8	95.5	―	97.9	96.6	―	98.0	96.7	―	98.0	97.1	―	**100**	98.1	―	**100**									
*G. japonicus*	12	97.7	―	98.0	96.9	―	98.2	97.8	―	98.1	97.9	―	98.2	98.2	―	**100**	98.5	―	**100**	**99.6**	―	**100**						
*G. kanchanaburiensis*	3	**99.2**	―	**99.2**	98.3	―	**99.5**	**99.4**	―	**99.4**	**99.5**	―	**99.5**	96.4	―	97.6	96.3	―	97.6	97.5	―	97.9	**100**	―	**100**			
*G. kondonii*	1	**99.2**			98.8	―	**100**	**100**			**99.9**			96.8	―	98.2	96.6	―	98.2	97.8	―	97.9	**99.4**	―	**99.4**	**100**		
*G. morbifer*	2	97.4	―	97.5	96.8	―	98.1	97.9	―	98.0	98.0	―	98.1	96.6	―	97.8	96.4	―	97.8	97.6	―	97.8	97.7	―	97.8	97.9	―	97.9
*G. oxydans*	79	96.8	―	**99.7**	95.7	―	**99.6**	96.8	―	**99.6**	96.9	―	**99.5**	96.5	―	**100**	96.2	―	**100**	97.4	―	**100**	96.6	―	**99.2**	96.5	―	97.9
*G. potus*	1	**99.7**			98.0	―	99.2	**99.1**			**99.2**			96.8	―	98.0	96.6	―	97.9	97.9	―	98.0	**99.1**	―	**99.1**	**99.1**		
*G. roseus*	5	**99.4**	―	**99.7**	97.8	―	**99.3**	**98.9**	―	**99.2**	**99.0**	―	**99.3**	96.7	―	98.1	96.6	―	98.1	97.8	―	98.4	**99.1**	―	**99.2**	98.9	―	99.2
*G. sphaericus*	5	**99.2**	―	**99.3**	**98.7**	―	**100**	**99.9**	―	**99.9**	**99.9**	―	**100**	96.8	―	98.2	96.7	―	98.1	97.9	―	98.2	**99.4**	―	**99.5**	**99.9**	―	**99.9**
*G. thailandicus*	11	97.5	―	97.8	96.5	―	98.0	97.6	―	98.0	97.7	―	98.0	98.0	―	**99.9**	98.2	―	**100**	**99.2**	―	**99.9**	97.3	―	97.6	97.6	―	97.9
*G. vitians*	1	**99.5**			98.0	―	99.1	**99.1**			**99.1**			96.8	―	97.9	96.8	―	98.2	97.9	―	98.3	**99.2**	―	**99.2**	**99.0**		
*G. wancherniae*	2	98.1	―	98.1	97.1	―	98.2	98.2	―	98.2	98.2	―	98.2	97.5	―	**99.2**	97.9	―	**99.2**	98.9	―	**99.2**	97.9	―	97.9	98.2	―	98.2

	*G. morbifer*			*G. oxydans*			*G. potus*			*G. roseus*			*G. sphaericus*			*G. thailandicus*			*G. vitians*			*G. wancherniae*		
*G. aidae*																								
*G. albidus*																								
*G. cadivus*																								
*G. cerevisiae*																								
*G. cerinus*																								
*G. frateurii*																								
*G. japonicus*																								
*G. kanchanaburiensis*																								
*G. kondonii*																								
*G. morbifer*	**100**	―	**100**																					
*G. oxydans*	96.6	―	97.9	96.4	―	**100**																		
*G. potus*	97.6	―	97.8	96.9	―	**99.9**	**100**																	
*G. roseus*	97.7	―	97.9	96.9	―	**100**	**99.5**	―	**99.9**	**99.7**	―	**100**												
*G. sphaericus*	98.0	―	98.1	96.9	―	**99.5**	**99.1**	―	**99.2**	**98.9**	―	**99.3**	**99.9**	―	**100**									
*G. thailandicus*	97.2	―	97.7	97.1	―	**100**	97.7	―	98.0	97.7	―	98.1	97.7	―	98.0	**99.6**	―	**100**						
*G. vitians*	97.8	―	98.0	97.1	―	**99.8**	99.7			**99.8**	―	**99.9**	**99.0**	―	**99.1**	97.8	―	98.2	**100**					
*G. wancherniae*	97.6	―	97.6	97.7	―	**99.2**	98.2	―	98.2	98.3	―	98.4	98.2	―	98.2	98.7	―	**99.0**	98.4	―	98.4	**100**	―	**100**

* Values ≥ 98.7 % (in bold) exceed the generally accepted species‐level threshold for 16S rRNA gene sequence identity

Rectangles highlight pairs that cross the 98.7 % threshold—either exceeding it between distinct species or falling below it within the same species.

The 16S rRNA genes of *Gluconobacter* exhibited exceptionally high sequence conservation across the genus. Pairwise comparisons among 184 sequences revealed overall identity values ranging from 93.38% to 100% (Supplementary Table S4). When restricted to sequences assigned to validly named species (as defined by genome‐based clustering at ≥ 95% ANI), identity values ranged from 96.3% to 100% (Table 3).

Most species, including *G. aidae, G. albidus, G. cadivus, G. cerevisiae, G. kanchanaburiensis, G. kondonii, G. potus, G. roseus, G. sphaericus*, and *G. vitisans*, showed pairwise identities exceeding the commonly accepted 98.7% species threshold in nearly all combinations (Chun et al. 2018), indicating that these taxa cannot be clearly distinguished based on 16S rRNA sequences alone. In contrast, the relationships among *G. cerinus, G. frateurii*, and *G. japonicus* displayed a contradictory pattern: while some interspecific comparisons exceeded the 98.7% cutoff, several intraspecific pairs fell below it.

*G. oxydans* also exhibited threshold‐crossing relationships with almost all other species except *G. kondonii* and *G. morbifer*, consistent with the presence of *G. thailandicus*‐related strains identified in the ANI analysis. Together, these findings reveal a paradoxical pattern of “overall conservation yet taxonomic inconsistency,” demonstrating that the 16S rRNA gene, despite its utility for genus‐level assignment, lacks the resolution necessary for accurate species‐level classification within *Gluconobacter*. In subsequent analyses, 74 unique, non‐redundant sequences were used.

### 3.3 Phylogenetic Relationships and Variable‐Region Characteristics

**Figure 1:**
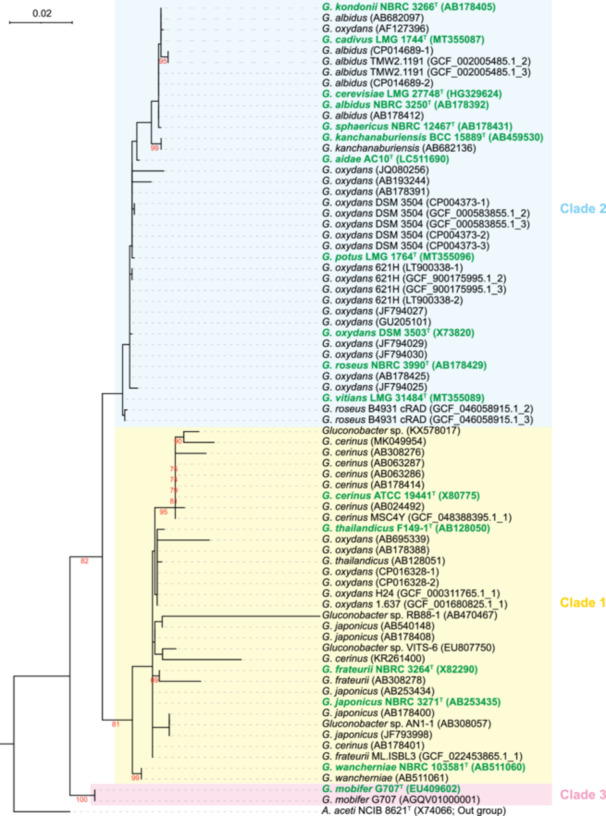
Maximum‐likelihood phylogeny inferred from 1546 bp 16S rRNA gene sequences of *Gluconobacter* species and related taxa. Three major clades (Clades 1–3) were consistently recovered, corresponding to distinct phylogenetic lineages within the genus. Type‐strain sequences are highlighted in green. Bootstrap support values (≥ 70%) are indicated at nodes and were calculated from 100 pseudo‐replicates. *Acetobacter aceti* NCIB 8621 ^T^ (X74066) was used as the outgroup. The scale bar represents 0.02 nucleotide substitutions per site.

To evaluate the phylogenetic resolving power of the 16S rRNA gene, a maximum‐likelihood tree was constructed using the 74 unique sequences (Figure 1). Three major phylogenetic clusters (corresponding to clades 1–3 in Figure 1) were reconstructed. Marked incongruence was observed between species names and phylogenetic clustering within *Gluconobacter*. Multiple type strains failed to form single, species‐specific clades. For instance, sequences assigned to *G. oxydans, G. frateurii*, and *G. japonicus* were dispersed across several branches. This indicates that sequences sharing the same species designation do not constitute monophyletic lineages and that the 16S rRNA gene provides inconsistent phylogenetic signals. In addition, some phylogenetic clusters spanned multiple species boundaries: a subset of *G. oxydans* sequences clustered near *G. thailandicus* or *G. frateurii*, whereas certain *G. thailandicus* entries were embedded within the *G. oxydans* clade. Furthermore, intra‐specific variation overlapped extensively with inter‐specific divergence, particularly in *G. oxydans* and *G. cerinus*. These findings further confirm that, despite its high conservation, the 16S rRNA gene alone is insufficient to delineate species boundaries within *Gluconobacter*.

**Figure 2:**
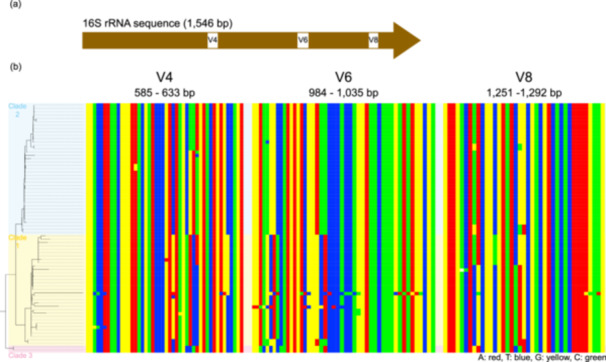
Sequence variation in the V4, V6, and V8 regions of the 16S rRNA gene among *Gluconobacter* species. (a) Schematic representation of the *Gluconobacter* 16S rRNA gene (1,546 bp) showing the positions of the hypervariable regions V4, V6, and V8. (b) Alignment of representative *Gluconobacter* 16S rRNA gene sequences highlighting the three variable regions (V4: 585–633 bp; V6: 984–1035 bp; V8: 1251–1292 bp). Nucleotides are color‐coded as follows: A (red), T (blue), G (yellow), C (green). The alignment is arranged according to the maximum‐likelihood tree (left). Sequence divergence is most pronounced in the V4, V6, and V8 regions, corresponding to the main phylogenetic clades (Clades 1–3). Conserved regions were omitted for clarity.

To further characterize sequence conservation and the distribution of nucleotide variability, the hypervariable regions (V1–V9) of representative *Gluconobacter* strains were compared. The majority of nucleotide substitutions were concentrated in the V4, V6, and V8 regions (Figure 2), while the remaining regions (V1‐3, V5, V7, and V9) were highly conserved. Even within these variable regions, no clear clade‐specific patterns were observed, and differences between groups were limited to only a few nucleotides. This analysis corroborates that the *Gluconobacter* 16S rRNA gene—despite minor variability within certain hypervariable regions—possesses limited discriminatory power for phylogenetic resolution at the species level.

## 4 Discussion

Importantly, the aim of this study is not to propose taxonomic reclassification but to quantitatively evaluate the correspondence between 16S rRNA similarity and genome‐wide divergence under conditions where ribosomal operons are fully resolved. Since its introduction into bacterial systematics (Fox et al. 1977), the 16S rRNA gene has served as a primary marker for prokaryotic taxonomy. In the present study, we evaluated 16S rRNA gene conservation and taxonomic resolution within currently available complete genomes of *Gluconobacter*. Consistent with previous observations (Yamada et al. 2011), 16S rRNA sequences showed extremely limited divergence, resulting in substantial overlap between intra‐ and interspecific similarities. Such patterns reflect strong functional constraints and slow evolutionary rates, explaining why 16S rRNA sequences remains suitable for genus‐level identification but fails to delineate closely related species.

ANI analysis further revealed that *G. oxydans* comprises multiple distinct genomic lineages, with some strains clustering more closely with *G. thailandicus* (Matsutani et al. 2014). While such polyphyly had been suggested previously (Matsutani et al. 2014; Tanasupawat et al. 2004; Yamada et al. 2011), our study directly links this genomic heterogeneity with the near‐identity of 16S rRNA sequences. Even hypervariable regions (V4, V6, V8) showed minimal differences and lacked lineage‐specific signatures, underscoring the intrinsic limitations of 16S rRNA‐based species classification. Because 16S cannot reliably separate species (Janda and Abbott 2007; Johnson et al. 2019), its use for species‐level assignments carries a substantial risk of misclassification. Such reliance may contribute to outdated nomenclature and obscure true genomic relationships. This limitation is not unique to *Gluconobacter* but has been widely reported across diverse bacterial taxa, where high 16S rRNA similarity masks substantial genome‐level divergence and complicates species delineation (Chun et al. 2018; Jain et al. 2018). Genome‐based metrics such as ANI or dDDH should therefore be used for definitive identification, while 16S rRNA gene sequences are more suitable for initial broad‐level screening.

The implications for microbiome studies are considerable. Similar discrepancies between marker‐based classification and underlying genomic or functional diversity have been reported in microbiome studies, where reliance on 16S rRNA can obscure true taxonomic and functional variation (Tanno et al. 2022). Our results suggest that reports of “*G. oxydans*” in 16S rRNA‐based surveys may represent mixtures of multiple species complexes that cannot be resolved at the species level. Consequently, genus‐level reporting (e.g., *Gluconobacter* sp.) is a more reliable and conservative practice. Additional quality controls—such as operon‐length filtering—may improve accuracy but cannot overcome the fundamental limits of the 16S rRNA gene. Overall, our findings indicate that the current *Gluconobacter* taxonomy does not fully reflect genomic structure. Although our study does not aim to formally revise the taxonomy of the group, the results clearly highlight inconsistencies between current species assignments and genomic relationships. *G. oxydans* may require taxonomic re‐evaluation, and boundaries with neighboring species warrant further assessment. Future work should incorporate genome‐based species boundaries, develop validated single‐gene markers (e.g., *rpoB*, *groEL*), and refine database annotations such as SILVA and GTDB. Expansion of genome‐based analyses using additional high‐quality genome assemblies will be important to further refine these observations and extend them across the full diversity of the genus.

In conclusion, while the 16S rRNA gene has played a central historical role in bacterial systematics, it shows inherent limitations in *Gluconobacter*. Its extreme sequence conservation prevents reliable inference of species boundaries, even when genome‐level differentiation is substantial. By providing a quantitative comparison of 16S rRNA gene diversity and genome‐based divergence within currently available complete genomes, this study clarifies why 16S rRNA sequences cannot support species‐level taxonomy in this group. Moving forward, an accurate and reproducible taxonomy for *Gluconobacter* will require a genome‐based framework supported by curated reference databases, validated single‐gene markers, and a careful re‐examination of species‐level boundaries.

## Author Contributions

**Shintaro Maeno:** conceptualization, methodology, data curation, writing – review and editing, writing – original draft, visualization, formal analysis, project administration. **Naoya Kataoka:** writing – review and editing, formal analysis, investigation. **Minenosuke Matsutani:** methodology, writing – review and editing, formal analysis. **Toshiharu Yakushi:** writing – review and editing, formal analysis, supervision. All authors read and approved the final manuscript.

## Funding

The authors have nothing to report.

## Ethics Statement

The authors have nothing to report.

## Consent

The authors have nothing to report.

## Conflicts of Interest

The authors declare no conflicts of interest.

## Supporting information


**Table S1**: Complete genome information of *Gluconobacter* strains used in this study. List of *Gluconobacter* strains analyzed in this study with complete genome sequences, including assembly accessions, genome sizes, and gene counts.

**Table S2**: 16S rRNA gene sequences and accession numbers of type strains of *Gluconobacter* species used in this study. 16S rRNA gene sequences and accession numbers of *Gluconobacter* type strains used for phylogenetic analysis. *Acetobacter aceti* NCIB 8621^T^ (X74066) was included as an outgroup.

**Table S3**: Pairwise nucleotide differences among 16S rRNA gene copies across *Gluconobacter* complete genomes. The table shows the pairwise number of nucleotide differences among 16S rRNA genes detected in each genome assembly. Rows and columns correspond to individual 16S rRNA gene copies, labeled as “Genus_species_GCF_ID_copyNumber”. Zero indicates identical sequences, whereas larger values indicate the number of base differences. This dataset illustrates both intra‐genomic heterogeneity (within the same strain) and inter‐species divergence among *Gluconobacter* members.

**Table S4**: Pairwise sequence identity (%) among 184 16S rRNA gene sequences associated with *Gluconobacter*. This table summarizes pairwise percent identities among 184 16S rRNA gene sequences associated with the genus *Gluconobacter*. The dataset includes all 16S copies extracted from 12 complete genomes, 18 type‐strain reference sequences, and curated entries retrieved from the SILVA database. All sequences longer than 1,350 bp were aligned against each other using BLASTN (v2.14.1) with parameters ‐task blastn ‐evalue 1e‐10 ‐max_hsps 1 ‐max_target_seqs. 1000. Percent identity values were derived from the top‐scoring HSPs in each pairwise comparison. Diagonal values indicate self‐comparisons (100%), whereas off‐diagonal values represent sequence similarity either within genomes (intragenomic) or between different strains or species (intergenomic/interspecific).

## Data Availability

The data that support the findings of this study are available in NCBI GenBank at https://www.ncbi.nlm.nih.gov/genbank/. These data were derived from the following resources available in the public domain: ‐ SILVA database, https://www.arb-silva.de/. ‐ NCBI GenBank, https://www.ncbi.nlm.nih.gov/genbank/.
